# African Savanna-Forest Boundary Dynamics: A 20-Year Study

**DOI:** 10.1371/journal.pone.0156934

**Published:** 2016-06-23

**Authors:** Aida Cuni-Sanchez, Lee J. T. White, Kim Calders, Kathryn J. Jeffery, Katharine Abernethy, Andrew Burt, Mathias Disney, Martin Gilpin, Jose L. Gomez-Dans, Simon L. Lewis

**Affiliations:** 1 Department of Geography, University College London, Gower Street, WC1E 6BT, London, United Kingdom; 2 Center for Macroecology, Evolution and Climate, University of Copenhagen, Universitetsparken 15, DK-2100 Copenhagen, Denmark; 3 Agence Nationale des Parcs Nationaux, BP 20379, Libreville, Gabon; 4 Institute de Recherche en Ecologie Tropicale, BP13354, Libreville, Gabon; 5 School of Natural Sciences, University of Stirling, FK9 4LA, Stirling, Scotland, United Kingdom; 6 Earth Observations, Climate and Optical Group, National Physical Laboratory, Hampton Road, Teddington, Middlesex, TW11 0LW, United Kingdom; 7 Laboratory of Geo-Information Science and Remote Sensing, Wageningen University, Wageningen, The Netherlands; 8 UK NERC National Centre for Earth Observation (NCEO), Michael Atiyah Building, University of Leicester, University Road, LE1 7RH, Leicester, United Kingdom; 9 School of Geography, University of Leeds, LS2 9JT, Leeds, United Kingdom; Technical University in Zvolen, SLOVAKIA

## Abstract

Recent studies show widespread encroachment of forest into savannas with important consequences for the global carbon cycle and land-atmosphere interactions. However, little research has focused on *in situ* measurements of the successional sequence of savanna to forest in Africa. Using long-term inventory plots we quantify changes in vegetation structure, above-ground biomass (AGB) and biodiversity of trees ≥10 cm diameter over 20 years for five vegetation types: savanna; colonising forest (F1), monodominant Okoume forest (F2); young Marantaceae forest (F3); and mixed Marantaceae forest (F4) in Lopé National Park, central Gabon, plus novel 3D terrestrial laser scanning (TLS) measurements to assess forest structure differences. Over 20 years no plot changed to a new stage in the putative succession, but F1 forests strongly moved towards the structure, AGB and diversity of F2 forests. Overall, savanna plots showed no detectable change in structure, AGB or diversity using this method, with zero trees ≥10 cm diameter in 1993 and 2013. F1 and F2 forests increased in AGB, mainly as a result of adding recruited stems (F1) and increased Basal Area (F2), whereas F3 and F4 forests did not change substantially in structure, AGB or diversity. Critically, the stability of the F3 stage implies that this stage may be maintained for long periods. Soil carbon was low, and did not show a successional gradient as for AGB and diversity. TLS vertical plant profiles showed distinctive differences amongst the vegetation types, indicating that this technique can improve ecological understanding. We highlight two points: (i) as forest colonises, changes in biodiversity are much slower than changes in forest structure or AGB; and (ii) all forest types store substantial quantities of carbon. Multi-decadal monitoring is likely to be required to assess the speed of transition between vegetation types.

## Introduction

There is growing evidence that woody encroachment into savannas is occurring worldwide [1,2,3]. This has been attracting attention, because if woody encroachment is widespread, it has important consequences for the global carbon cycle and land-atmosphere interactions. For example, a recent modelling study by Poulter et al. [4] highlights that since 1981, a six per cent expansion of vegetation cover over Australia was associated with a fourfold increase in the sensitivity of continental net carbon uptake to precipitation. Deforestation exceeds forest gains across much of the tropics [5], and some have argued that there is a bias towards the detection of deforestation as opposed to woody encroachment or recovery [3]. In Africa, evidence of woody encroachment is scattered but widespread, covering a range of ecosystems and rainfall levels: from West Africa through Central Africa, Ethiopia and South Africa [3,6].

In the Congo basin, the second largest block of contiguous tropical forest after the Amazonian basin, the interface between tropical forest and savanna is a structurally and floristically diverse mosaic of vegetation types, with forest penetrating into the savanna as gallery forests along river banks and as forest patches on plateaus [7]. Within Central Africa, savannas are likely maintained by a combination of precipitation, soil characteristics and anthropogenic disturbance such as fire and clearance for grazing, agriculture and timber [8,9]. In the Congo basin, it has been suggested that forest is expanding into savannas because of urban-migration and a consequent reduction in fire frequency [10], or driven by higher atmospheric CO_2_ concentration [4,11], similarly to the more positive conditions for tree growth documented in intact forests [12,13].

While the number of studies assessing woody encroachment in Africa has increased in the past few years (see [3] for a review), most focus on detecting forest expansion (tree cover change, tree density or Leaf Area Index, LAI), and not the assessment of the characteristics of these different forests as they undergo forests succession, often because studies use mostly remotely sensed data, thus subtle changes within forests are difficult to assess. Hence, there remains much uncertainty on forest dynamics and succession from savanna to old-growth forest in the tropics [14]. In fact, within the Congo basin, the few studies available on forest dynamics assess recovery after logging [15,16,17], or within intact forests [12] with few studies addressing savanna-forest succession, although studies from central Gabon provide a notable exception [18,19,20,21].

In the coming decades, African forests are predicted to experience profound climatic changes with increased temperature, alteration of rainfall patterns and possibly longer dry seasons [13,22,23]. Thus a better understanding of ecosystem functioning within different forest types is urgently required, as well as predictions of how they may respond to climatic changes [13,24,25]. Furthermore, assessing the carbon stored in different forests and soils is necessary to participate in schemes to reduce emissions from deforestation and degradation in the tropics, (e.g. the UN Framework Convention on Climate Change REDD+ initiative), as well as Nationally Determined Contributions for countries that include decreases in emissions from land-use change [26].

Soils are also an important carbon pool. In some tropical environments, such as in the African miombo woodlands, soil carbon stocks are greater than those aboveground [27]. Sources of soil organic carbon (SOC) include root turnover, leaf litter, and woody debris in forests, or grasses necromass in savanna. Vegetation types with high net primary production (NPP), such as old-growth forests are expected to have high soil carbon [28]. Apart from soil carbon, soil chemical and physical conditions can also constrain the amount of biomass stored in tropical forests, with different forest types often linked to different soil types [9,29,30].

While studies of the differing vegetation types in the putative succession from savanna to forest in central Gabon have been published [18,19], no studies have considered long-term phytodemographic change within different forest types in central Gabon, or to our knowledge Central Africa. Here we use long-term inventory plots to quantify the changes in AGB, vegetation structure and biodiversity of five vegetation types over 20 years. These five types, in probable successional order, run from savanna to colonising forest to monodominant Okoume forest, young Marantaceae forest (still Okoume monodominant overstorey) and mixed Marantaceae forest (mixed species overstorey; [18,19]. The mixed forest is not the end of the succession, mixed forest without abundant Marantaceae is likely to follow mixed Marantaceae forest [18], but this does not occur in the immediate vicinity, so was not sampled. After assessing whether soil properties were driving any of the different vegetation communities, we identified two key questions. First, do changes in AGB, vegetation structure and biodiversity across the five vegetation types follow the pattern expected of increasing AGB, followed by a decline in stem density in mature forest following self-thinning, and steadily increasing diversity? Second, after 20 years, has any vegetation type altered enough in structure, AGB or diversity to be re-classified as another vegetation type in the hypothesised successional sequence?

## Materials and Methods

### Study area

**Fig 1:**
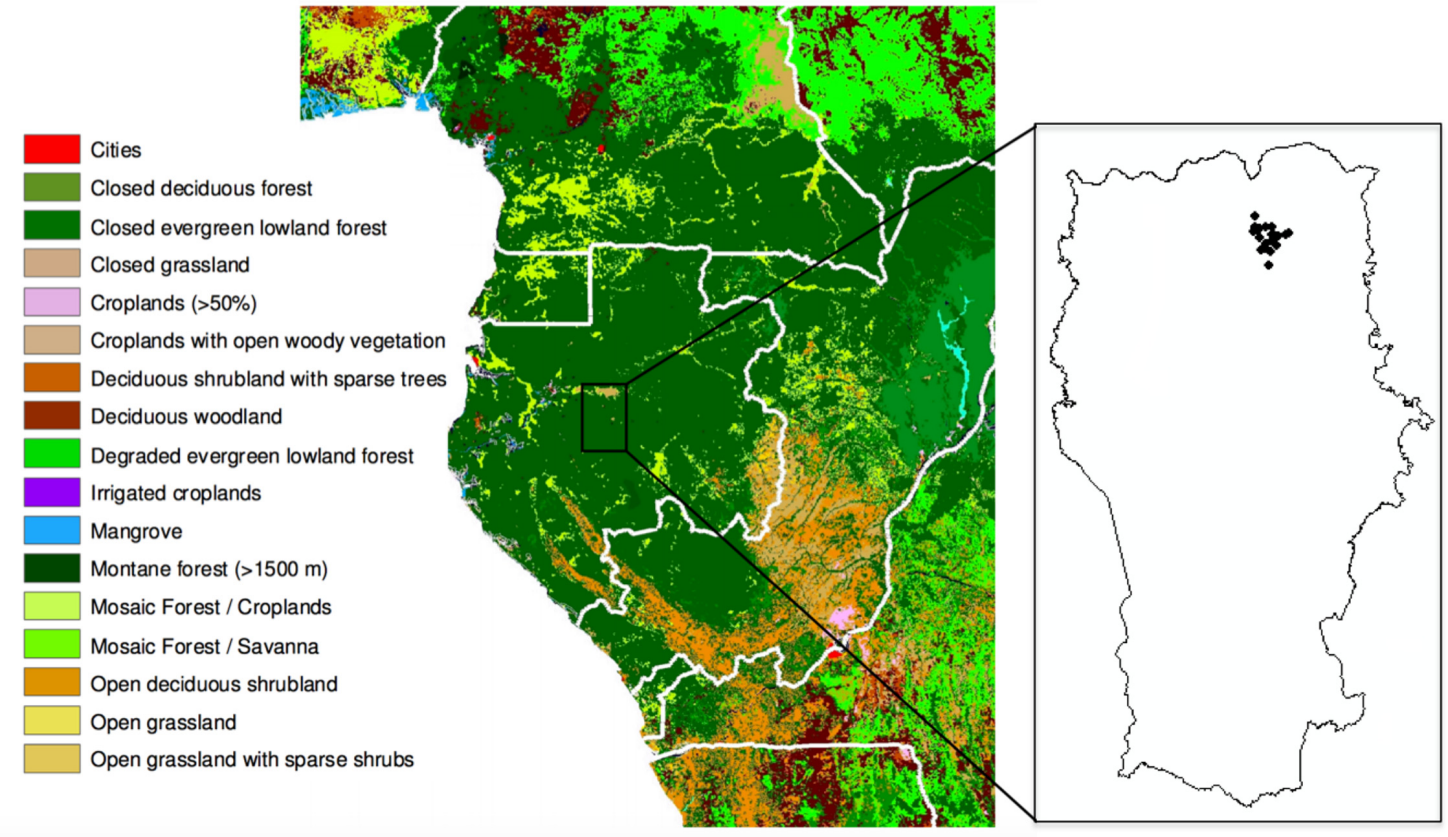
Lopé National Park (LNP) and plots sampled in the north of the park characterised by a savanna-forest mosaic. Map showing the location of the field site within a landcover map for the year 2000 [78] adapted from Mitchard et al. [21].

The study area was Lopé National Park (LNP) located in central Gabon (0° 10’S 11° 35’ E). Established as a wildlife reserve in 1946, it became a National Park in 2007 covering 4960 km^2^, one of the country’s largest protected areas. While most of the park is closed-canopy tropical rainforest, the north of the park is characterised by a savanna-forest mosaic (Fig 1), a remnant of the landscape that dominated much of the Congo basin during the Last Glacial Maximum (LGM) [31]. During the LGM savanna covered the majority of LNP, whereas increased precipitation since the beginning of the Holocene when much of central Africa became wetter, thus causing an expansion of forest to cover nearly the whole area, with forest continuing to expand into the savannas today, combined with waves of human activity and abandonment since the expansion of Bantu farming culture [19,31]. The savanna that currently remains is maintained by a combination of human burning and the rain-shadow of the Massif du Chaillu, which reduces rainfall to 1500 mm yr^−1^ in the north of the park compared to about 2500 mm yr^−1^ in the south [32].

A diversity of vegetation types have been described within LNP [18,19]. Here we focus on five distinct major vegetation types that occur close to the forest-savanna boundary: savanna, colonising forest, monodominant Okoume forest, young Marantaceae forest and mixed Marantaceae forest. Although the concept of succession from savanna to old-growth forest has been debated, particularly for African savannas (e.g. [33], within LNP, in absence of disturbance, savannas (S) become colonising forest (F1), then monodominant Okoume forest (F2), then young Marantaceae forest (F3), the mixed Marantaceae forest (F4), and eventually mature old-growth forest [19]. In this study we consider these five distinct vegetation types which appear to show successional stages, ending with mixed Marantanceae forest <700 years old, as reported by White [18,19].

**Fig 2:**
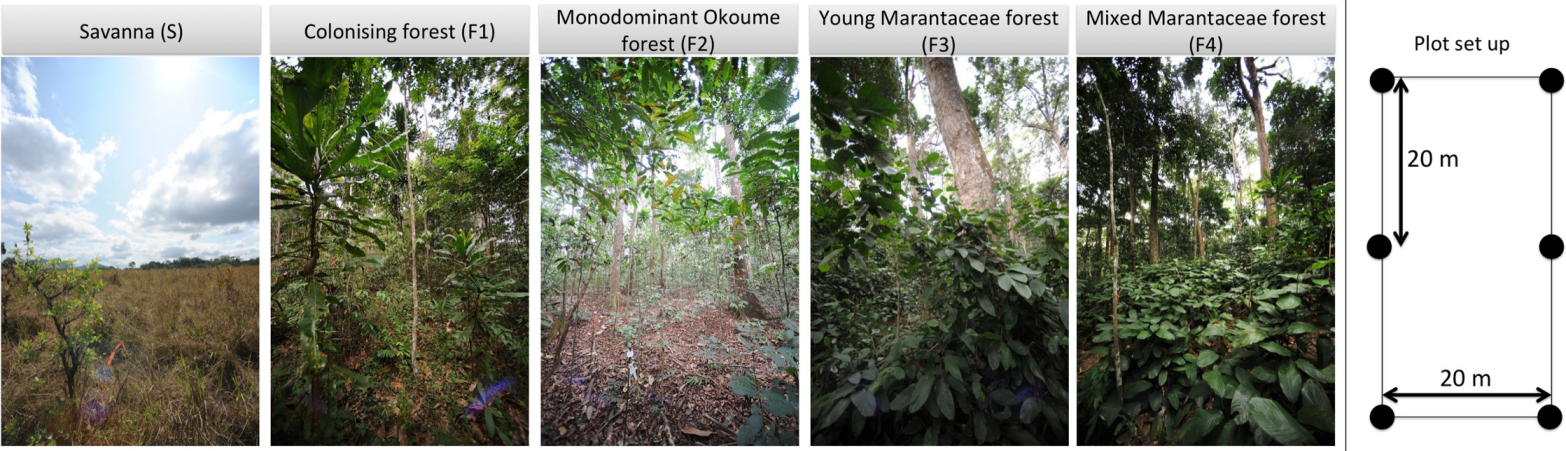
**Different forest types (left) and plot set up (right)**.

Savannas of LNP are tree species-poor, with no *Acacia* spp. and dominant species including *Crossopteryx febrifuga*, *Bridelia ferruginea*, *Sarcocephalus latifolius* and *Psidium guineensis*. Colonising forest is characterised by an open canopy, as trees are not sufficiently large and tall to fully meet one another (Fig 2), and the presence of heliophile species such as Okoume (*Aucoumea klaineana*, Burseraceae), *Lophira alata* (Ochnaceae) and *Sacoglottis gabonensis* (Humiriaceae). Monodominant Okoume forest (F2) is characterised by a closed canopy of *A*. *klaineana* trees of similar age and an open understory (Fig 2). Young Marantaceae forest (F3) consists of larger Okoume trees and a very distinctive understory dominated by a thick layer of herbaceous plants of the Marantaceae and Zingiberaceae families produced within light gaps created by falling trees, often of pioneer species (Fig 2). Mixed Marantaceae forest (F4) refers to a mature Marantaceae forest with very large trees emerging of a diversity of species coupled with some areas of very low-stature vegetation, and an understorey dominated by Marantaceae and Zingiberaceae families. This has the highest tree species richness compared with the other forest types. Further details on the vegetation types of LNP and the different successional stages can be found in White [18,19] and White and Abernethy [32]. Although the term closed canopy is often defined as ‘areas where tree cover exceeds 40 per cent while the term open forest refers to areas where tree cover is between 10 and 40 per cent’ (see [34,35], White and Abernethy [32] refer to closed canopy as something > 60–70% canopy cover.

Even though occurrence/prevalence of tree harvesting inside National Parks and other protected areas is a widespread phenomenon in Africa, this is typically not the case in Gabon. The Lopé reserve experienced low-level selective logging, usually for Okoume, prior to becoming a National Park, between 1965 and 1980 [18]. Firewood harvesting does not occur as the population density is very low adjacent to the park. Savanna fires have occurred for at least the timespan of human occupation, at least 40,000 years. A human population expansion following the arrival of Bantu farmers ~4,500 years ago increased the number of fires, while a human population crash ~1,400 years ago reduced the numbers of fires [36]. Recent historical fires were set by local people, but since 1993 a fire management plan has been in place to help maintain the regionally-rare savanna ecosystems [18,37].

### Field measurements

Five plots in each vegetation type were installed in 1993 [18], each 20 × 40 m with similar altitude and topography, using standard field inventory methods (see Fig 2). Plots were established in areas with the best known burn history, including the use of aerial photographs, and were classified into different vegetation types according to canopy cover, tree height, species’ diversity and dominance, presence/absence of a thick layer of herbaceous plants and vertical forest structure [18]. Overall, savannas were open grassland with sparse trees <4m height and low tree species’ diversity. F1 had more trees but < 70% canopy cover, trees >4m and low tree species’ diversity. F2 had >70% canopy cover and canopy height of 15-20m, and were dominated by *A*. *klaineana*, F3 had <70% canopy cover, this was dominated by *A*. *klaineana*, and a thick layer of herbaceous plants of the Marantaceae and Zingiberaceae families. F4 also had a thick layer of herbaceous plants of the Marantaceae and Zingiberaceae families, plus much larger trees, higher canopy cover and high tree species diversity. Plots were re-measured in 2013, except one F1 plot which could not be relocated in 2013 due to tag loss. In all plots all living free-standing woody stems ≥10 cm diameter at 1.3 m along the stem from the ground (or above buttresses/deformities if present) were measured and stems were identified to species where possible. In 2013 tree height was measured using a laser hypsometer (Nikon Forestry Pro).

In three plots per vegetation type, soil samples were also collected in 2013 at depths of 0-5cm, 5-10cm, 10-20cm, 20-30cm and 30-50cm. The litter layer was excluded. These were collected at the centre of the plot using soil sampler that does not disturb the soil (Eijkelkamp Agrisearch Equipment BV, Giesbeek, The Netherlands). Samples were air-dried.

In two plots per vegetation type, vertical forest structure was assessed in 2013 using novel 3D terrestrial LiDAR measurements [38,39]. This TLS (Terrestrial LiDAR Scanner) data were acquired with the RIEGL VZ-400 3D instrument (RIEGL Laser Measurement Systems GmbH, Horn, Austria). Full hemispherical scan data were collected at scan resolution of 0.06° in the azimuth and zenith directions. Each plot had six scan locations, following a systematic 20 × 20 m sampling design (Fig 2). The scanner records multiple return data, with a maximum of four returns per emitted pulse, which improves vertical sampling in the upper canopy [40].

### Analysis of soil samples

On arrival in the laboratory all soil samples were oven dried to constant mass at 40°C. Methods followed tropical standard methods, see Quesada et al. [41] for full details. Briefly, sieving, weighing and subsampling, particle size (sand/silt/clay) and pH were carried out following ISRIC [42]. Cation exchange capacity (CEC) and weatherable elements (Ca, Mg, P, Fe, Al, Na, etc.) sample preparation also followed ISRIC [42], and were measured using Inductively Coupled Plasma Optical Emission Spectrometry (ICP-OES, Perkin Elmer Optima 5300DV). For CEC the modified silver–thiourea method [43] was used. Bulk density (BD, gcm^−3^) followed Rowell [44] and carbon and nitrogen content were analysed following the manufacturers’ recommendations for ground fine material using the Vario Micro Gas Combustion Analyser (Elementar Co.). Phosphorous was analysed by sequential extraction following Tiessen and Moir [45]. All soil samples were analysed at the soil laboratory of the University of Leeds.

The soil organic carbon pool (SOC) in each layer, in Mg ha^-1^, was estimated using the equation SOC = C*BD*V in which C, is the proportion of a given mass of soil that is carbon, BD is the bulk density of the soil (mass per unit volume), in Mg m^−3^, and V the volume (m^3^) of soil.

### Fire history of the studied plots

As fire frequency may be an important factor determining woody encroachment we assessed fire history for each of the plots. First, information on planned and recorded fires between 1995 and 2008 [37], and from 2009–13 (LNP managers) was gathered. Second, burned area data from the official MODIS MCD45A1 Collection 5.1 product [46] and the MCD64A1 product, including thermal anomalies [47] were obtained. Monthly burned area data was extracted for each plot, between April 2000 and July 2013. During the managed burning period (1995–2013), all savanna plots were burned between five and 19 times, but no fire was recorded entering F1-F4 stages of forest. Prior to 1993 it is presumed that unmanaged fires affected savanna plots and possibly F1 plots, but not F2-F4 plots.

### Data analysis

For each plot, we calculated Basal Area (BA); stem density, and BA-weighted wood mass density (WMD_BA_) following Lewis et al. [48]. For AGB the Chave et al. [49] equation including diameter, wood mass density (WMD) and tree height was used to estimate the AGB of each tree in the plot. Diameter was used as measured, unless a change in the point of measurement (POM) occurred. These tend to occur when trees grow fast and buttresses form, but the raising of the POM underestimates the true growth of the stem. In these cases we harmonize the two disjointed sets of growth measurements (from the original POM, and the new POM) by replacing the measured diameters with the mean of (1) the ratio of the original to the new POM diameter measurements (to standardize each diameter measurement to the height of the original POM), and (2) the ratio of the final to the original POM diameter measurement (to standardize each diameter measurement to the height of the final POM; [12,50]). The best taxonomic match of wood density to each stem was extracted from a global database [51,52] following Lewis et al. [48]. As 1993 field measures did not include tree height, a best estimate of the height in 1993 based on available data was used. First, following Feldpausch et al. [53] the relationship between tree diameter and height was established using a Weibull function [54] for each forest type separately. These relationships were then used to estimate heights for every tree in both 1993 and 2013 (H_weibull_). Then, for each tree, the difference between H_weibull_ (estimated height) in 2013 and field-measured height (H_real_) provides an offset from which to estimate height in 1993, by subtracting the offset from H_weibull_ in 1993 to obtain H_real_ in 1993

Stem density (number of trees ha^-1^) included all trees ≥10 cm diameter, while BA (sum of the cross-sectional area at 1.3 m, or above buttresses) was calculated in m^2^ ha^-1^. WMD_BA_ (the mean of the WMD of each stem weighted by its BA) was estimated as dry mass/fresh volume, in g cm^-3^. AGB change terms were divided into growth (gain in AGB due to tree growth and tree recruitment) and mortality (loss due to tree mortality) components. For AGB gains, we also added the productivity of newly recruited stems, *senus* Talbot et al. [50] using the 86^th^ percentile growth rate of stems from the same plot census in the 10–19.9 cm size class is used, since this provides the closest approximation of the mean growth of recruits. Stem turnover was calculated as the mean of the number of stems recruited and lost due to mortality, *sensu* Phillips and Gentry [55].

Three biodiversity metrics where calculated for 1993 and 2013: species richness, the Shannon index (*H’*) and the Bray-Curtis Index of dissimilarity (BC). Species richness was determined as total number of species observed in a given plot. *H’*, a measure of biodiversity calculated from the relative abundance of species in a community, was computed separately per each plot as:

$$H^{\prime }=\sum_{i=1}^{s}p_{i}\;\text{ln}\;p_{i}$$

where *p*_*i*_ = n_i_/N, n_i_ is the number of individuals present of species i, N the total number of individuals, and S is the total number of species.

The Bray-Curtis Index of dissimilarity (BC), used for comparing the dissimilarity and diversity of sample sets, was defined as:

$$BC=\frac{S_{i,j}}{S_{i}+S_{j}}$$

where S_i,j_ are the species found in both sample sets, S_i_ is the total number of species of sample set i, and S_j_ is the total number of species of sample set j. A value of BC = 1 indicates complete similarity, while BC = 0 indicates complete dissimilarity. As we wanted to establish if vegetation types were becoming more similar with increasing time, BC between a given vegetation type and the following vegetation type in the succession in 1993 was computed by combining all plots within each vegetation type in 1993. Then, BC between a given vegetation type in 2013 and the following vegetation type in the succession in 1993 was also computed.

In order to assess if species’ dominance changed, for each plot we also computed species dominance (in terms of % of BA) of two common light-demanding species: the above-mentioned *A*. *klaineana* (which forms monodominant stands in F2), and *L*. *alata*, a tree often found alongside *A*. *klaineana* in forest regrowth [32].

Vertical plant profiles of each plot were derived from terrestrial LiDAR through estimates of the vertically resolved gap fraction. Vertical plant profiles describe the plant area volume density (PAVD) as a function of canopy height and can be used to quantify the plant area index (PAI), but also to derive various canopy height metrics, see Calders et al. [56] for full details. The PAVD is defined as the plant area per unit crown volume (m^-1^) and the PAI is the total one-sided plant area per unit ground area [38]. Potential errors in vertical plant profiles related to terrain were corrected following Calders et al. [40]. Each forest plot was scanned from six locations and the plot vertical profiles were calculated by averaging the vertically resolved gap fraction of each individual scan. Savanna plots were not assessed with TLS since no trees with > 10 cm diameter were present thus no canopy was present.

R statistical software R v3.2.1 and RStudio v.0.99.447 were used for all statistical analyses [57]. A Bonferroni correction was applied when considering differences in soil characteristics amongst vegetation types. The study was carried out with permission as part of the government of Gabon national carbon inventory programme. The TLS data was collected under a CENAREST research permit and ANPN National Park entry permit. All plant species in the park are protected, with some protected throughout Gabon, and some on IUCN lists. Permits covered measurements of these trees as part of the study.

## Results

### Soil characteristics

**Fig 3:**
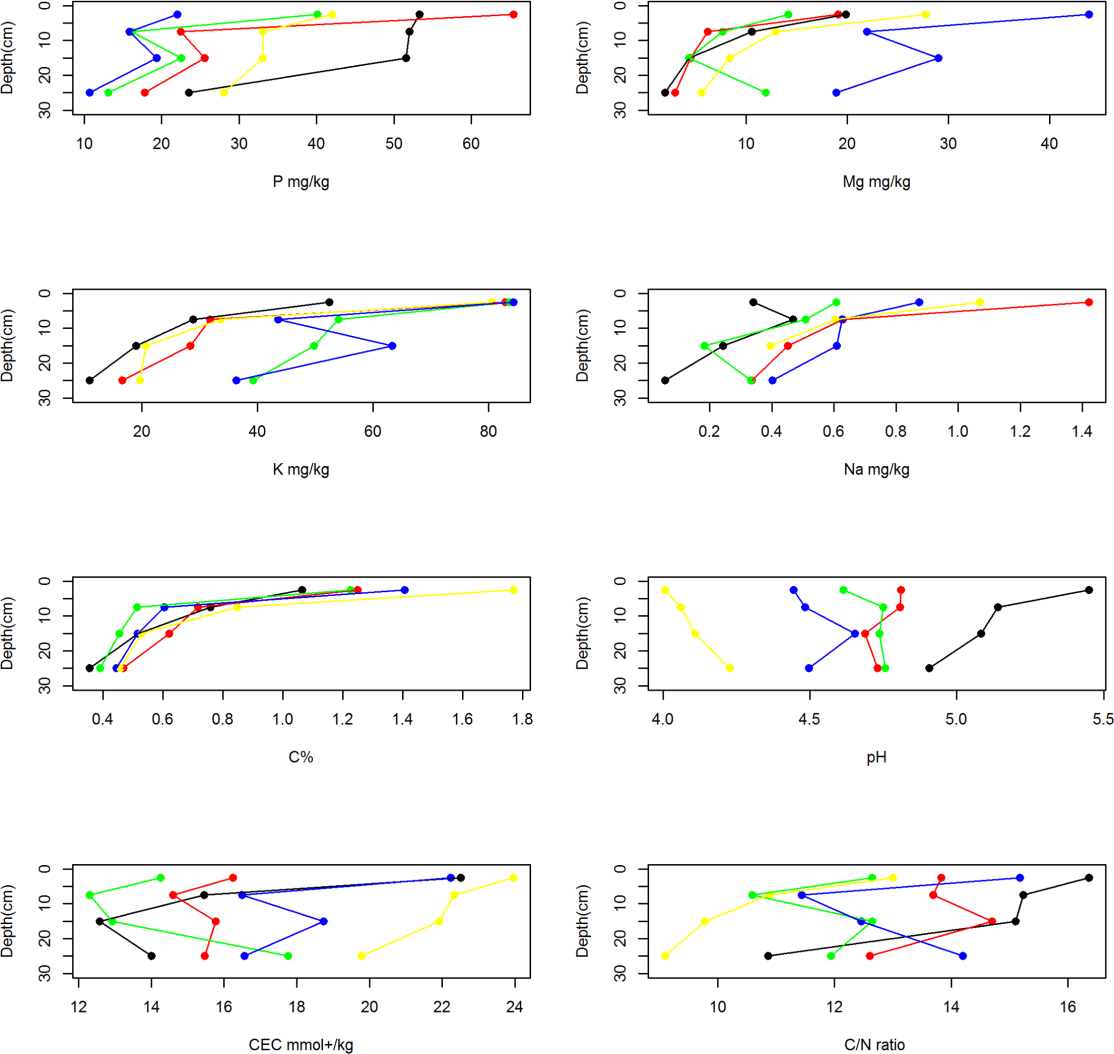
Changes in soil characteristics with increasing depth per forest type (mean plotted). Note that black dots refer to savanna, red dots: colonising forest (F1), green dots: monodominant Okoume forest (F2), blue dots: young Marantaceae forest (F3) and yellow dots: mixed Marantaceae forest (F4).

Only small differences in soil characteristics between vegetation types were observed, mostly in comparisons between the extreme ends of the putative succession: savanna and mixed Marantaceae forest (F4) soil (Tables 1 and 2). Overall, savanna soils had significantly higher pH and C:N ratio but lower cation exchange capacity (CEC) and aluminium than F4 forests while the other forest types had intermediate values (Tables 1 and 2). F4 forests also had significantly lower Bulk density (BD) than other forest types. No differences in C%, total C or total P between vegetation types were observed (Table 1). Few differences were noted between the soils beneath the three most mature forest vegetation types (F2, F3 and F4), although F4 forest had significantly lower pH, higher aluminium, and lower bulk density than F2 or F3 forest types. While P, Mg, K, Na and C% decreased significantly with increasing soil depth in all vegetation types, this pattern was not clear for soil pH, CEC and C:N ratio (Fig 3). With regard to micronutrients, savanna soils had significantly higher Mn than other vegetation types but lower Zn and Fe than F4 (Table 2). No other significant differences in micronutrients (Ba, Co, Cu, Mo, Ni) were observed.

**Table 1:** Soil characteristics, 0-30cm depth, per forest type, including soil pH, Carbon %, C:N ratio, effective cation exchange capacity (eCEC), Bulk Density (BD), soil carbon and total Phosphorus.

	pH (H_2_O)		C%		C:N ratio		eCEC mmol+/kg		BD g/cm3		Soil carbon MgC/ha		Total P mg/kg	
Savanna (S)	5.1 ± 0.3	a	0.67 ± 0.3	a	14.3 ± 2.4	a	14.4 ± 3.1	a	1.31 ± 0.06	a	23.7 ± 3.7	a	37.6 ± 28.1	a
Colonising forest (F1)	4.7 ± 0.15	b	0.76 ± 0.35	a	13.7 ± 1.3	ab	15.5 ± 3.4	a	1.32 ± 0.09	a	25.3 ± 2.1	a	32.8 ± 33.1	a
Monodominant Okoume forest (F2)	4.7 ± 0.2	b	0.64 ± 0.49	a	11.9 ± 2.0	bc	14.3 ± 4.8	a	1.34 ± 0.09	a	22.3 ± 8.5	a	22.9 ± 13.5	a
Young Marantaceae forest (F3)	4.5 ± 0.2	b	0.74 ± 0.45	a	13.3 ± 2.2	ab	18.5 ± 3.7	ab	1.33 ± 0.05	a	26.3 ± 6.7	a	16.9 ± 7.3	a
Mixed Marantaceae forest (F4)	4.1 ± 0.1	c	0.90 ± 0.57	a	10.7 ± 2.1	c	21.9 ± 4.5	b	1.21 ± 0.07	b	27.2 ± 4.4	a	34.0 ± 14.4	a

Different letters within a column (a, b, c) indicate significant differences between forest types at p<0.01.

**Table 2:** Soil characteristics, 0-30cm depth, per forest type, for several weatherable elements.

	Al^3+^ mg/kg		Ca^2+^ mg/kg		Mg ^2+^ mg/kg		K^+^ mg/kg		Na^+^ mg/kg		Mn mg/kg		Zn mg/kg		Fe mg/kg	
Savanna (S)	68.2 ± 39.6	a	108.2 ± 85.4	a	9.1 ± 10.4	a	27.8 ± 17.3	a	0.27 ± 0.42	a	26.4 ± 34.3	a	0.11 ± 0.03	a	5.5 ± 4.4	a
Colonising forest (F1)	121.3 ± 34	b	6.3 ± 8.41	a	8.2 ± 8.8	a	39.9 ± 37.6	a	0.7 ± 0.74	a	3.3 ± 3.4	b	0.34 ± 0.45	a	25.8 ± 13.1	a
Monodominant Okoume forest (F2)	87.6 ± 46.2	ab	46.5 ± 113.4	a	9.5 ± 7.4	a	56.7 ± 30.9	a	0.40 ± 0.29	a	8.3 ± 10.4	b	0.24 ± 0.17	ab	11.7 ± 9.2	a
YoungMarantaceae forest (F3)	106.9 ± 30.7	ab	56 ± 60.2	a	28.4 ± 24.2	b	56.9 ± 35.1	a	0.62 ± 0.27	a	4.3 ± 4.8	b	0.39 ± 0.46	ab	40.7 ± 19.1	b
Mixed Marantaceae forest (F4)	170.4 ± 42	c	18.5 ± 21.9	a	13.6 ± 9.32	a	38.6 ± 33.9	a	0.48 ± 0.54	a	4.5 ± 5.3	b	0.63 ± 0.33	b	45.1 ± 42.1	b

Different letters within a column indicate significant differences between forest types at p<0.01.

Soil C stocks at 0–30 cm depth ranged from 23.7 to 27.2 Mg C ha^-1^ (S and F4 respectively), but were not significantly different amongst vegetation types (Table 1). For depth 0-50cm, C stocks ranged between 31.6 and 41.1 Mg C ha^-1^ (S and F4 respectively). Macroscopic charcoal fragments were found in one F1 plot (5–10 cm) and three F4 plots (5–10 cm and 30–50 cm).

### Observations in 1993

In 1993, no tree >10 cm diameter was found in any savanna plot (trees <10 cm were observed). Colonizing forests (F1) had significantly less AGB, at 43 Mg dry mass ha^-1^, than the other forest types (>300 Mg dry mass ha^-1^, see Table 3). F1 forests also had significantly lower BA than other forest types. WMD_BA_ ranged between 0.45 and 0.64 (Table 3). Species richness ranged from 5.7 to 13.4 per 0.08 ha plot, and H' from 1.24 to 2.26, with only F1 forests being significantly different from F4 forests in terms of diversity metrics (Table 4). While F4 had lower *A*. *klaineana* dominance than F2 and F3 (5% compared with 50%), there were no differences in *L*. *alata* dominance amongst forest types (Table 4). Stem densities were lowest in F1 plots, highest in F2 plots and low in F3 and F4 plots.

**Table 3:** Above ground biomass (AGB in Mg ha^-1^), annual change (AGB change in Mg ha^-1^ year^-1^), changes in AGB related to losses from mortality (AGB Mort in Mg ha^-1^) and gains for recruitment (AGB Recr in Mg ha^-1^) and growth of surviving stems (AGB Grow in Mg ha^-1^), basal area (BA in m^2^ ha^-1^), stem density (S in number stems ha^-1^), wood mass density weighted by BA (WMD_BA_) in 1993 and 2013 per forest type.

	AGB 1993		AGB 2013		AGB change		AGB Mort		AGB Recr		AGB Grow		BA 1993		BA 2013		S 1993		S 2013		WMD 1993		WMD 2013	
Colonising forest (F1)	42.8 ±23.1	a	103.6 ± 35.5	a*	3.1 ± 1.5	a	14.1 ± 21.9	a	45 ± 17.8	a	+30.4 ± 20.7	a	6.27 ± 3.71	a	13.1 ± 5.7	a*	247.2 ± 123.8	a	506.5 ± 156.9	a*	0.64 ± 0.12	a	0.72 ± 0.05	a*
Monodominant Okoume forest (F2)	313.3 ± 45.1	b	388.7 ± 55.6	b*	3.8 ± 0.6	a	20.1 ± 8	a	15.4 ± 5.8	b	80 ± 10.6	b	32.6 ± 8.58	b	42.6 ± 11.5	b*	507.6 ± 62	b	505.4 ± 90.7	a	0.51 ± 0.18	a	0.52 ± 0.18	ab
Young Marantaceae forest (F3)	442.7 ± 130.1	b	448.6 ± 212.4	b	0.3 ± 4.8	a	91.1 ± 88.1	a	7.8 ± 3.9	b	89.2 ±29.6	b	46.4 ± 14.2	b	48.9 ± 21.9	b	395.2 ± 138.2	ab	415.4 ± 133.5	ab	0.45 ± 0.07	a	0.47 ± 0.05	b
Mixed Marantaceae forest (F4)	475.8 ± 229.1	b	495.1 ± 274.1	b	1.1 ± 5.6	a	67.8 ± 91.7	a	2.9 ± 2	b	84.3 ±34.6	b	39.0 ± 13.6	b	39.4 ± 14.4	b	325.4 ± 125.7	ab	282.8 ± 87.89	b	0.64 ± 0.05	a	0.64 ± 0.06	ab

Different letters within a column indicate significant differences amongst forest types at p<0.01.

* refers to significant differences between 1993 and 2013 at p<0.01 for that measure and forest type. As no savanna plot had any tree >10cm diameter in either 1993 or 2013, no further calculations were made for this vegetation type.

**Table 4:** Species richness (No spp), Shannon index (H’), *A*. *klaineana* dominance (in %BA), *L*. *alata* dominance (in %BA) and the Bray-Curtis (BC) Index of dissimilarity in 1993 and 2013 (compared with the following vegetation type in the succession in 1993) per forest type.

	No spp 1993		No spp 2013		H' 1993		H' 2013		*A*. *klaineana* 1993		*A*. *klaineana* 2013		*L*. *alata* 93		*L*. *alata* 2013		BC 1993	BC 2013
Colonising forest (F1)	5.7 ± 2.7	a	8.5 ± 2.5	a*	1.24 ± 0.37	a	1.26 ± 0.2	a	0		0		28.1 ± 33.6	a	43 ± 27.6	a*	0.24	0.29
Monodominant Okoume forest (F2)	9.6 ± 3.6	ab	10 ± 4	a	1.57 ± 0.48	ab	1.54 ± 0.59	ab	55.2 ± 31.5	a	54.3 ± 33	a	18.6 ± 25.5	a	19.3 ± 25.3	a	0.25	0.35
Young Marantaceae forest (F3)	9.6 ± 1.9	ab	10.2 ± 2.4	a	1.84 ± 0.23	ab	1.98 ± 0.21	ab	54 ± 17	a	47.6 ± 22.3	a	5.6 ± 3.9	a	8.5 ± 5.7	a	0.23	0.24
Mixed Marantaceae forest (F4)	13.4 ± 3.9	b	11.8 ± 3.2	a	2.26 ± 0.63	b	2.2 ± 0.51	b	4.1 ± 3.9	b	5.8 ± 5.3	b	6.1 ± 11.9	a	7 ± 12.4	a	na	na

Different letters within a column indicate significant differences between forest types at p<0.01.

* refers to significant differences between 1993 and 2003 at p<0.01 for that measure and forest type. As no savanna plot had any tree >10cm diameter in either 1993 or 2013, no further calculations were made for this vegetation type.

### Observations in 2013

In 2013, as in 1993, no tree >10 cm diameter was found in any savanna plot. After 20 years F1 and F2 forests had gained AGB, but the rank AGB order did not change. F1 forests had significantly less biomass than the other forest types (100 Mg ha^-1^ versus > 400 Mg ha^-1^, see Table 3). Although F1 forests had significantly increased stem density and BA since 1993, in 2013 stem density was significantly higher than F4 forests, while BA was still significantly lower than F4 forests (Table 3). In 2013 F1 forests had significantly increased in WMD_BA_, and had significantly higher WMD_BA_ than F3 (Table 3), possibly due to the significantly increased dominance of *L*. *alata*, which has a particularly high wood density. With regard to biodiversity, only F1 had increased in species richness, but the H' pattern remained the same, with F1 forests continuing to be significantly different from F4 forests (Table 4). While *L*. *alata* dominance had increased in F1 forests, *A*. *klaineana* dominance patterns had not changed (Table 4).

### Changes within vegetation types

#### Savanna (S)

No tree >10 cm diameter was found in any savanna plot in either 1993 or 2013. The woody structure of these plots is characterised by small trees <10cm basal diameter, of two species only (*Crossopteryx febrifuga* and *Psidium guineensis*), which are small stature trees that rarely attain 10 cm diameter. Neither of these species was found in any forest plot.

#### Colonising forests (F1)

**Fig 4:**
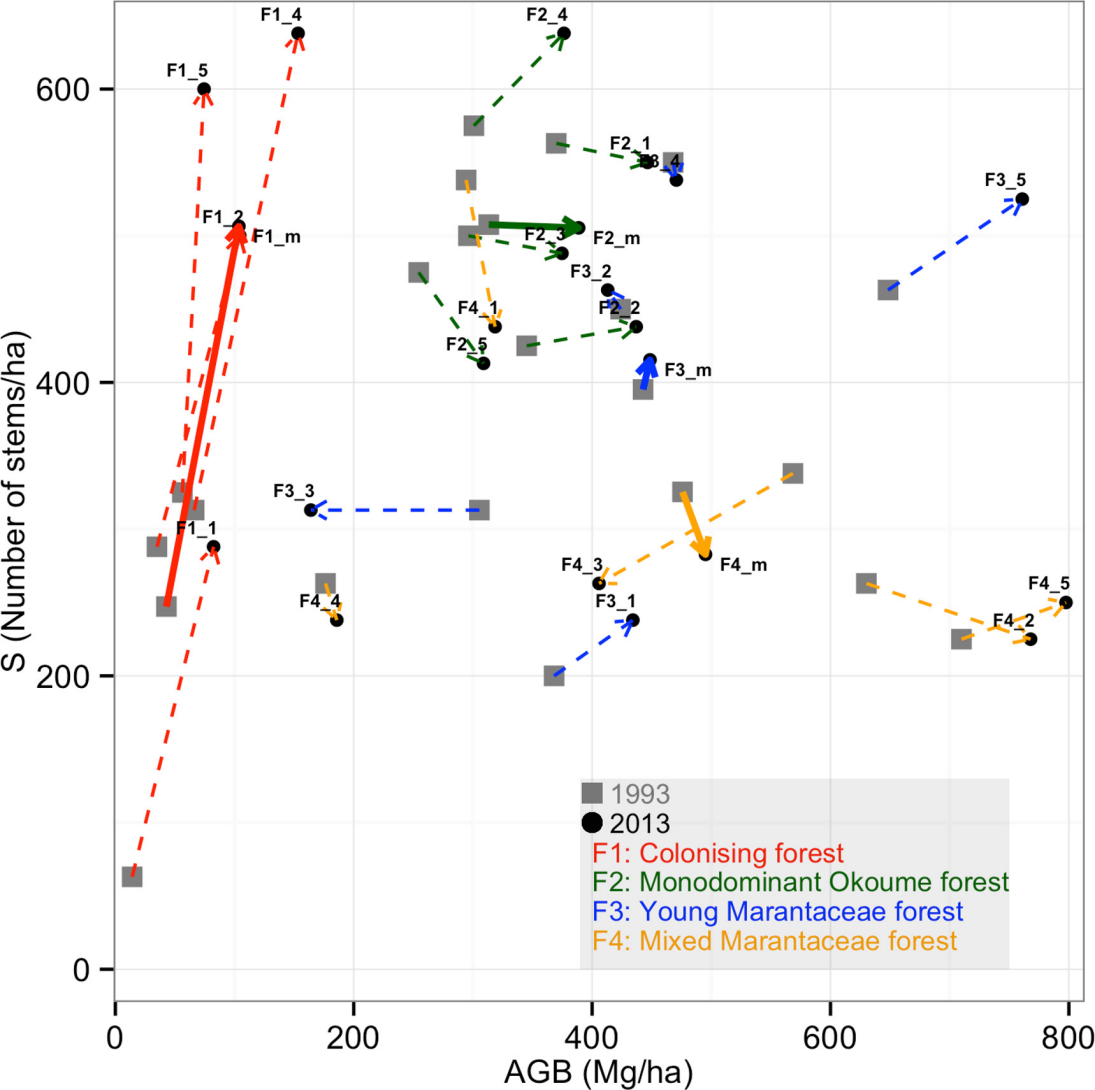
Changes of plots over time with regard to AGB and SD for each single plot (dotted line), and the mean per forest type (continuous line).

**Fig 5:**
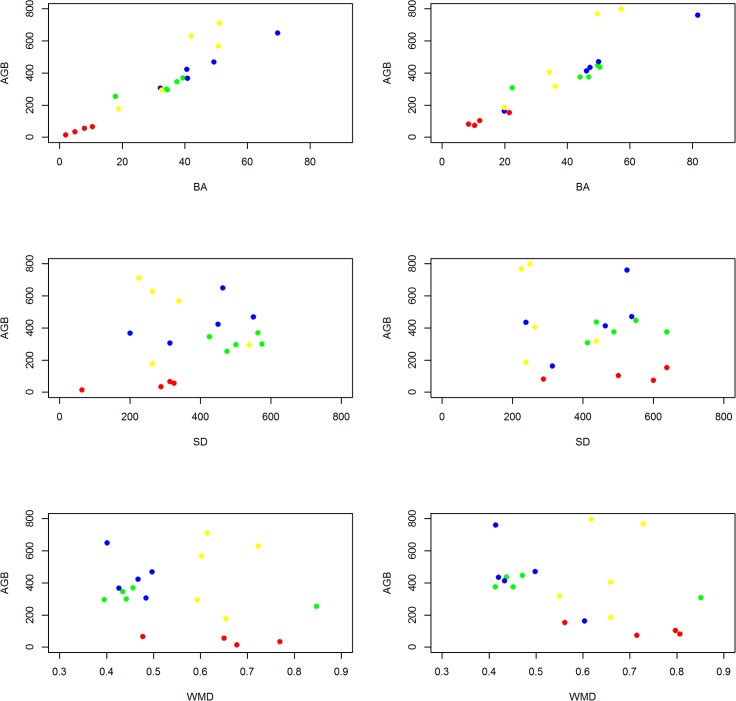
**Above ground biomass (AGB in Mg ha^-1^), in relation to basal area (BA in m^2^ ha^-1^), stem density (SD in number stems ha^-1^) and wood mass density weighted by BA (WMD) in 1993 (left) and 2013 (right).** Red: colonising forest (F1), green: monodominant Okoume forest (F2), blue: young Marantaceae forest (F3) and yellow: mixed Marantaceae forest (F4).

Between 1993 and 2013 F1 forests significantly increased in AGB, BA, stem density and WMD_BA_ (Table 3, Figs 4 and 5). Although species richness and *L*. *alata* dominance significantly increased, H' did not significantly change (Table 4). No *A*. *klaineana* was recruited in any F1 plots, likely because this species is strongly shade intolerant and highly fire sensitive.

#### Monodominant Okoume forests (F2)

Between 1993 and 2013, F2 forests also significantly increased in AGB and BA but not in stem density (Table 3). WMD_BA_, species richness, H’, *A*. *klaineana* or *L*. *alata* dominance did not significantly change (Tables 3 and 4). Although 20 years saw an increase in the AGB and BA of F2, none of the F2 plots could be classified as F3 in 2013. For F2 plots, high values of AGB were related to middle values of BA, stem density and low WMD_BA_ (Fig 5).

#### Young Marantaceae forests (F3)

F3 forests were very stable across the 20 years: they did not significantly increase in AGB, BA or stem density between 1993 and 2013 (Table 3). In fact, two plots lost AGB due to large trees dying, F3.3 lost two trees >50cm diameter and one >1m diameter, while F3.4 lost two trees >50cm diameter (see Fig 4). Neither species richness, H' nor WMD_BA_ changed significantly during this period (Tables 3 and 4). F3 plots had similar values of AGB, BA, stem density and biodiversity to F4 plots, both in 1993 and 2013. F3 plot *A*. *klaineana* dominance in 1993 remained in 2013 (Table 4). Twenty years did not significantly change F3 forests; therefore, none of F3 plots from 1993 could be classified as F4 in 2013. For F3 plots, as for F2 plots, high values of AGB were also related to middle values of BA, stem density and low WMD_BA_ (Fig 5).

#### Mixed Marantaceae forests (F4)

F4 forests were very stable across the 20 years: they did not significantly increase in AGB, BA or stem density (Table 3). One F4 plot lost 30% of its 1993 AGB due to two trees >70cm diameter dying. Neither species richness, H' or WMD_BA_ changed significantly during this period. For F4 plots, high values of AGB were related to high BA and WMD_BA_ but low stem density (Fig 5).

### Comparing putative successional stages

Overall, F1 and F2 forests increased in AGB, mainly as a result of adding stems (recruitment) in the case of F1 forests, or increased BA in the case of F2 forests. Some plots of F3 and F4 increased in AGB while some decreased.

Relative change in stem density and AGB over time was different depending on forest type (Fig 4). Considering changes in stem density and AGB together, in Fig 4, F1 forests mainly moved along the y-axis (larger increases in stem density relative to AGB) while F2 forests moved along the x-axis (larger increases in AGB relative to stem density). F3 and F4 plots were more scattered (some had high or relatively low stem density and AGB) and changes occurred in different directions, as some plots lost AGB due to large trees falling, and others recovered from local disturbance events. Eight plots of the twelve plots which increased in AGB (of F2, F3 and F4) decreased in stem density, suggesting a tendency towards stand self-thinning (Fig 4).

When the relationship between AGB and other parameters is considered, in general, F4 plots have higher values of AGB because of high BA and WMD_BA_ but lower stem density, while F3 and F2 forests have higher values of AGB as a result of having intermediate values of BA and stem density, and lower values of WMD_BA_ (Fig 5).

Annual change in AGB (Mg dry mass ha^-1^ year^-1^) was not significantly different between the forest types, due to high variation within forest types (Table 3). Changes in AGB related to losses (from mortality) were not significantly different between forest types. However, changes related to gains (growth of surviving stems and recruitment) were significantly lower in F1 compared to the other forest types, with F1 forests having significantly higher AGB from recruitment of new stems than other forest types (Table 3).

Regarding diversity, only F1 significantly increased in species richness over the 20 years. F1 plots also increased in *L*. *alata* dominance, but not in H'. No significant biodiversity-related changes were observed for F2, F3 or F4 plots. The Bray-Curtis Index of dissimilarity between a given vegetation type and the following vegetation type in the succession in 2013 was slightly higher than in 1993 (Table 4).

### Vertical structure

**Fig 6:**
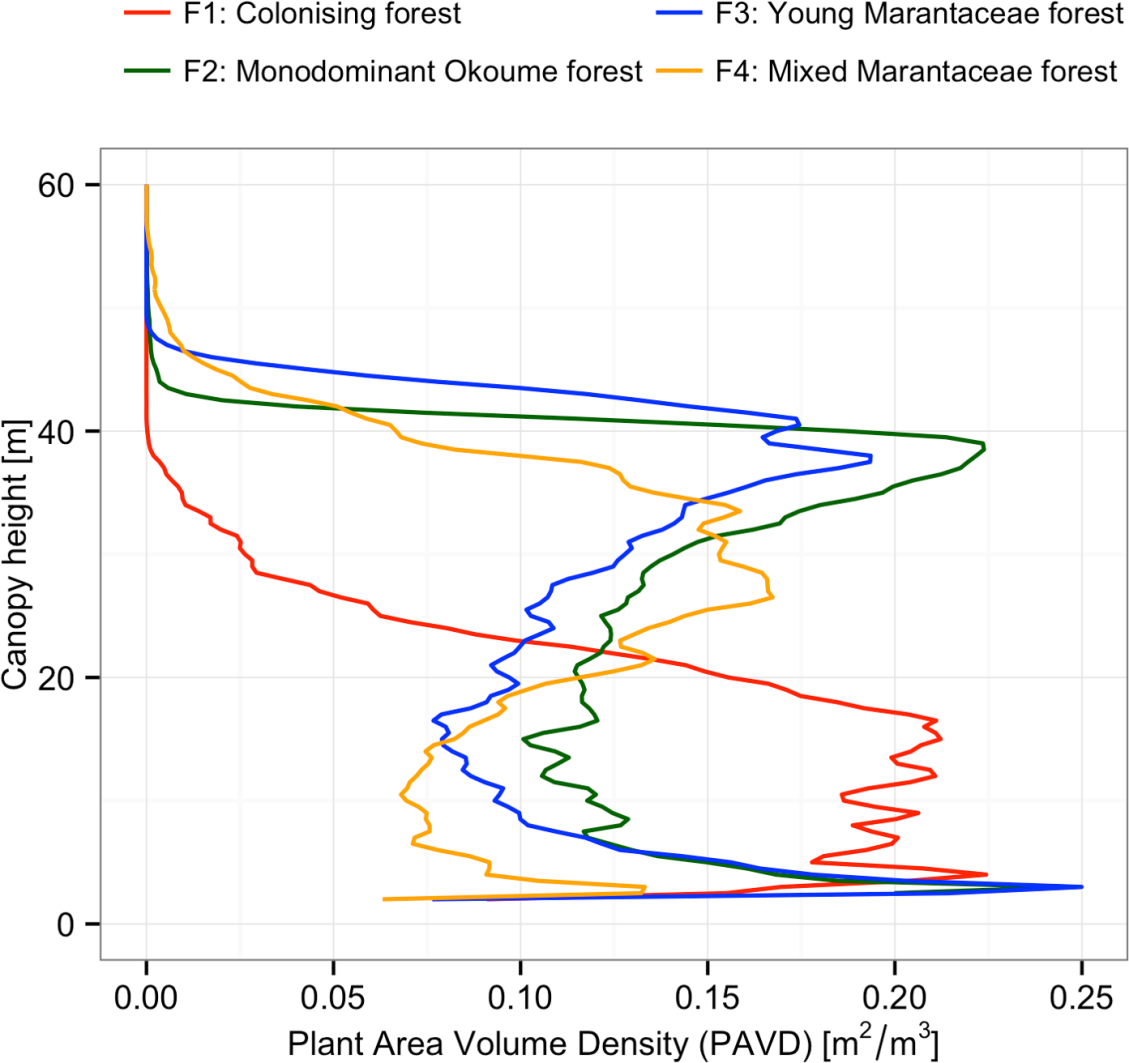
Mean vertical profiles per vegetation type.

Vertical plant profiles derived from TLS data were different depending on forest type (Fig 6). Plant area volume density (PAVD) was highest for F1 forests at 20 m, and there were few trees >30 m. PAVD had two peaks for F2 and F3 forests, at 3 m and 38.5 m. The peak in the upper canopy in F2 forests was larger than for F3 forests (canopy of most *A*. *klaineana*). Both F2 and F3 forest types show a second, lower large peak at 3 m due to thick Marantaceae understory. F4 forests also showed a bimodal vertical plant profile; with an upper canopy PAVD peak around 26.5 m, and lower peak at 3m. Some variation around each profile was found, related to the small number of plots sampled (two per vegetation type).

### Fire history of the plots

Observations by the authors showed that for the period 1995–2013, the savanna plots were each burned between five and 19 times. No fires were detected in any other plot. No fires were detected in any of our savanna plots by the two MODIS burned area products used between 2000 and 2013. The discrepancy is likely due to dry-season cloudiness meaning that data was missing for most of the fire-prone months each year, thus the use of MODIS products to infer fire impacts for this region is not further recommended.

## Discussion

### Soil characteristics

Savanna soils had significantly higher pH and C:N ratio but lower CEC and aluminium than other forest types, which is likely to be related to fire frequency in this vegetation type. Burning tends to clear vegetation and then stimulate fresh vegetation growth, and is known to increase soil pH (related to Ca and Mg being released from organic matter being burned) but decrease organic C, N, exchangeable aluminium and sulphur, the latter being volatilized by fire [58].

With regard to soil C stocks at depths of 0–30 cm, there was no clear successional gradient, as values ranged from 23.7 to 27.2 Mg C ha^-1^ and were not significantly different amongst vegetation types. The values in F1-4 are much lower soil C stocks than those reported in the literature (Table 5), including very recently collected soil carbon data at the same study site [59], the latter probably related to local heterogeneity at sampling sites and the method these authors used to estimate BD. This is consistent with soil maps of Gabon showing Lopé savannas and adjacent forest having extremely old and weathered soils [60]. However, it should be noted that: (i) few studies have assessed soil C stocks in Africa; (ii) great variation has been reported in such studies that have been undertaken; and (iii) these studies often report values for different depths (0-20cm, 0-30cm, 0-50cm, 0-100cm, 0-200cm, which make comparisons difficult, see Table 5). The lack of difference in soil C stocks amongst vegetation types is on the one hand not surprising, as the geology and soil types do not differ. On the other hand, elsewhere significant differences between both savannas and nearby forests, and between different forest types have been documented. For example, Coetsee et al. [61], in a study of soil C change related to woody plant encroachment in South Africa, reported that forests contained significantly more soil carbon than adjacent grassland savannas for 0–100 cm depth. However, unfortunately, these authors do not provide absolute values to be compared with our study. Differences in soil C stocks between different forest types have also been reported: Djomo et al. [62] show mixed forests having greater stocks than Caesalpinioideae rich forests (Table 5), although this may be also due to differing soil types. Overall, the low organic matter inputs into these soils over long periods of time–as the forests are all young (<700 years [19])–likely explains the low and similar C stocks in the soils underlying these forests.

**Table 5:** Mean soil Carbon stocks in this study and several studies available in Africa.

	mean C stocks (Mg C/ha)	depth assessed
*forests*		
This study in LNP F1	25.3	0-30cm
This study in LNP F2	22.3	0-30cm
This study in LNP F3	26.3	0-30cm
This study in LNP F4	27.2	0-30cm
Cameroun, Djomo et al. [62] (Caesalpinioideae rich forest)	44	0-30cm
Cameroun, Djomo et al. [62] (mixed species forest)	89	0-30cm
Cameroun, Njomgang et al. [79]	79	0-50cm
Gabon, Gautam and Pietsch [80]	66	0-20cm
	186	0-200cm
Ghana, Chiti et al. [81]	151	0-100m
Ghana, Saiz et al. [82]	40	0-30cm
	120	0-200m
*open savanna grassland*		
This study in LNP	24	0-30cm
Saiz et al. [82]	11	0-30cm

### Changes in AGB and vegetation structure

First, it should be noted that our sampling method only included trees ≥10 cm diameter, and savanna trees in our plots did not reach this size. Jeffery et al. [37] reported that some savanna patches protected from fire, located near the forest edge thickened rapidly over a 15-year period to become classified as colonising forest. Savanna plots sampled in this study were all burnt at least five times, suggesting that fire has played a role in preventing rapid transition to F1 type forest during this period.

Comparing the forest types along the putative successional gradient young (F1) forests increased in stem density while oldest forests (F4) tended to have a net loss of individuals (a decrease in stem density). These changes broadly follow the pattern expected from the literature [18,19,63], and can be seen in Fig 4.

AGB significantly increased over time in F1 and F2 forests. In F1 forests, greater AGB was related to an increase in stem density and BA while in F2 forests it was only related to an increase in BA. Indeed, when the canopy is still open (F1), recruitment of trees is high (e.g. between 240 and 520 individuals ha^-1^ were recruited into F1 plots over the period 1993–2013) and mortality is low, but with time, as the canopy closes (as in F2) and as competition for light and resources increases, forests tend towards self-thinning with recruitment reducing and mortality increasing. Even though self-thinning is a debated topic in forest ecology (see [64]), it is a common view that F2 forests (monodominant Okoume forests) follow this pattern, as Fuhr et al. [65] reported: ‘BA increases but stem density decreases with stand age’. Because trees become increasingly prone to disturbance with increasing age [66], and F2 forests are close to a single cohort stand, the death of old trees creates large gaps that can take many years to refill. This more open canopy is typical of young Marantaceae forests (F3), where more light reaches the forest floor, which is then colonised by ground-level Marantaceae and Zingiberaceae plants.

The stability over 20 years in terms of structure, AGB and diversity of F3 suggests either that this successional type is very long-lived, or that this forest type might not be an intermediary stage towards F4. It has long been accepted that F3 forests were a preliminary stage of F4 forests, mainly found at the forest edge physically located between F2 and F4 forests and in areas with high large mammal density especially gorillas and forest elephants [19,67]. However Tovar et al. [68] point out that Marantaceae forest might not be a successional stage, either following fire, savanna colonisation or post-agriculture regeneration [19], but may be a final stage in its own right. They found that the establishment of Marantaceae forests may also be associated with frequency of fires in forest rather than just savanna conversion to forest. Tovar et al. [68] also suggest that the mechanisms behind the maintenance of Marantaceae forest are more related to the opening of the canopy rather than to the establishment of the Marantaceae species themselves. Our results cannot distinguish between these alternative hypotheses. But they suggest that at a local scale the various paths that F3 forests follow can be quite different from one another. Some plots might be ‘trapped’ as young F3 forest, becoming a final stage in itself, due to forest ‘engineering’ in areas of high large mammal density; while other F3 plots might tend towards F4 and then old-growth forest. It should be noted that Marantaceae forests are not found in certain areas, such as coastal Gabon, where monodominant Okoume forests evolve directly to resemble the surrounding mixed forest [65]. More research is needed on the successional pathways of Marantaceae forests.

The amount of change in structure, AGB and diversity after 20 years was low. Even though the changes were greater at the beginning of the succession, no F1 or F2 plot could be classified as the following forest type in the succession. Forest recovery is expected to be faster in areas close to remnant forest patches, as recovery speed is considered to depend heavily on seed dispersal [14], although local climate conditions and soils might also be important. In our study area forest patches are close to areas of colonizing forest (often < 1 km) and animal dispersers are common. Nevertheless, we only observed a ‘fast’ increase of AGB for F1 and F2 forests, and even then little change in tree species diversity was seen. Forest recovery from direct human impacts is often assessed via changes in tree canopy cover (e.g. [69]), and indeed, F2 forests have a closed canopy while F1 do not. It has been reported that the succession (first stages) is more rapid during the colonisation of cultivated lands in the coastal area of Gabon than during the colonisation of paleo-climatic savannas in the LNP [65]. While it has been estimated that in Coastal Gabon it takes about 40 years to reach the monodominant Okoume forest stage [65], in LNP it has been predicted to take 100 years (AGB increase of 3 Mg dry mass ha^-1^ year^-1^), and F3 or F4, even longer. However, if biodiversity is also taken into account, it would take much longer as no *A*. *klaineana* was recruited in 20 years in any F1 plot, and this species is what defines F2 forests. Thus, given the potential 80–100 year transition from F1 to F2, it is not surprising that we did not document an F1-F2 transition in our 20 year study.

This is the first report on the use of TLS to obtain vertical plant profiles of different tropical forests types. Our vertical structure results from the different forest types support previous work in that (i) canopy height increases along the hypothesised succession, (ii) vertical forest structure is different amongst forest types, and (iii) the Marantaceae understory layer can be very thick in F3 [19]. Tropical forests, with high biodiversity and complex vegetation structure are often a challenge to classify. The use of TLS has potential for including objectively measured structural characteristics in vegetation classification, and if combined with botanical knowledge may be a powerful tool. We suggest TLS is therefore likely to prove a useful additional tool to analyse tropical forest structure. Furthermore, it can efficiently quantify relatively small changes in vegetation structure change [56]. Of course, vegetation varies so scanning many plots within a forest type will be required to capture robust patterns and differences amongst forest types.

### Changes in biodiversity and wood density

Significant biodiversity changes over time were only documented for species richness and *L*. *alata* dominance for F1. This supports the hypothesis that changes in biodiversity are much slower, occurring over centuries, compared to changes in forest structure or AGB occurring over decades [70]. No *A*. *klaineana* was recruited in any F1 plot in 20 years and in F2 *A*. *klaineana* density did not alter over 20 years. *A*. *klaineana* is a long-lived early-successional light demanding species mainly found in Gabon, Equatorial Guinea and Congo [71]. It is able to colonize open spaces, thus can form monodominant stands [72] and even-aged stands, and persists in mixed forests as other species are recruited. In our study *A*. *klaineana* in F2 forests were about 50% of BA, and appeared to be fairly even-aged stands. In F3 forests, *A*. *klaineana* trees were larger and less abundant, implying a maturing stand. *A*. *klaineana* comprised only about 5% of BA in F4. *A*. *klaineana* cannot resist fire. Fire, even once in 20 years, will be likely to differentially remove *A*. *klaineana* from a colonising stand (F1) and set the emerging forest on a successional track that will not include F2 or F3 stages as we describe them, but will have alternate successional stages. Overall, as forest type changes observed were slow, the sequence we see may be a very slow succession.

Our results for WMD_BA_ may appear surprising, as successional change in species are expected to result in an increase in WMD_BA_ when pioneer species are replaced with slower growing mixed forest species [27]. However, it should also be considered that savanna species often have high wood density, related to resistance to fire. In this study only F1 had significantly greater WMD_BA_ than F3, and this was related to *L*. *alata* dominance in F1, and the dominance of large *A*. *klaineana* trees in F3. *L*. *alata* has paradoxically high-density wood (0.897 g cm^-3^), unlike most light-demanding species which tend to have a light wood (e.g. *A*. *klaineana* = 0.378 g cm^-3^; [52]). *L*. *alata* heavy wood is linked to its fire-resistance. Although we did not find any *L*. *alata* in our savanna plots, single isolated stems of *L*. *alata* are also occasionally found in savannas. The mixed forest plots WMD_BA_ is typical of Central African tropical forests [48].

### Implications for management

The different forest types studied here each store considerable amounts of carbon as AGB (47% tropical forest AGB is carbon, [73]). F2, F3 and F4 forests had 182, 210 and 232 Mg C ha^-1^ respectively, which is similar to the mean value of African closed canopy forests, at 203 Mg C ha^-1^, and substantially higher than Amazonian values [48]. Furthermore, F1 and F2 increased in carbon stocks over the 1993–2013 period, mirroring wider patterns of increasing AGB in African forests [12, 16]. As many REDD+ carbon projects are 25–30 years long, protecting F1 or F2 forests might seem to be a relatively easy and/or cost-effective way to not only maintain but also increase carbon stocks on the land. Alternatively, F2 forests could provide timber, taking pressure off older more diverse forests. *A*. *klaineana* is the main timber export from Gabon (82% of the total timber production, see [74]). It has been suggested that these monodominant stands are not in equilibrium, and therefore that selective logging, with the consequent small scale canopy gaps, can be a way to promote a sustainable population for this species [75]. Although F2 forests store important quantities of carbon, and they increase their carbon stocks over time, their timber value is highest, therefore it might be more economically viable (and ecologically relevant for the species) to exploit F2 if this spares other more ecologically valuable forests from degradation.

Marantaceae forests (F3 and F4) sometimes considered degraded forest, are often assigned low conservation and research priority [76]. However, this forest type not only has great importance to several flagship species (gorillas, forest elephants) as it provides a dependable year-round supply of vegetative food [76], they also store large quantities of carbon per unit area. Thus this forest type, which may have been much more extensive in the past [19], may require active management to maintain areas of F3 and F4 forests [19,68]. However, how exactly to best undertake interventions to maintain such a system is currently unknown. Continued monitoring and research to ascertain if F4 is a long-term deflected succession is important.

The fire management plan was designed to reduce rates of forest expansion into the savanna to maintain a diversity of habitats in the forest-savanna transition zone [37]. This is important to maintain the ecologically distinct flora of the central Gabon savannas. One further aim of this was to encourage the seasonal use of savanna’s by large mammals as part of plans for the further development of tourism in and around LNP. Our results suggest that the current fire management programme has been sufficient to prevent savanna plots from converting to colonising forest in the 20 year study period. However, it should be noted that here we only report trees ≥10 cm diameter, thus woody thickening of savannas or compositional changes were not assessed. Jeffery et al. [37], using fixed-point photomonitoring methods, reported savanna thickening and forest expansion in certain parts of LNP. Veenendaal et al. [77] highlight that once subordinate woody canopy layers are taken into account, a less marked transition in woody plant cover across the savanna–forest species discontinuum is observed compared to that inferred when trees ≥10cm diameter are considered. Future work should assess these smaller stems and shrubs.

## Conclusions

To our knowledge, this is the first study assessing long-term phytodemographic changes over time along the succession in the Central African savanna-forest mosaic. Observed changes in AGB and vegetation structure followed our hypothesised directions, but the rate of change was found to be, overall, slow, especially with regard to changes in biodiversity and species’ dominance. After 20 years no plot could be classified as having moved to the next stage in our putative succession of forest types. Despite a lack of change, our study highlights the high carbon storage in AGB in these forests. Additional long term monitoring is required to better understand forest dynamics in Central Africa. Ideally, this will include TLS to provide precise and accurate structural parameters of each forest types and how these are changing.

Soil properties differed only between savanna and mixed Marantaceae forests, likely due to the effects of fire frequency on savanna soils. However, soil carbon stocks did not differ amongst any of the vegetation types, and different forest types did not occur on different soil types. Forest plots had much lower soil carbon stocks than other African forests.

Overall, the fire management plan to keep some areas of LNP as open savanna is maintaining savanna. Our documented increases in AGB across F1 and F2 forests suggest that carbon stocks may be increasing in LNP.

## References

[1] Archer S, Boutton TW, Hibbard KA (2001) Trees in grasslands: biogeochemical consequences of woody plant expansion In Global biogeochemical cycles in the climate system, eds. Schulze ED, Harrison S, Heimann M, Holland E, Lloyd J, Prentice I, Schimel D (CA: Academic Press, San Diego), 115–133.

[2] Naito AT, Cairns DM (2011) Patterns and processes of global shrub expansion. Prog Phys Geogr 35: 423–442.

[3] Mitchard ETA, Flintrop CM (2013) Woody encroachment and forest degradation in sub-Saharan Africa’s woodlands and savannas 1982–2006. Phil Trans R Soc B 368: 20120406 10.1098/rstb.2012.0406 23878342 PMC3720033

[4] Poulter B, Frank D, Ciais P, Myneni RB, Andela N, Bi J, et al (2014) Contribution of semi-arid ecosystems to interannual variability of the global carbon cycle. Nature 509: 600–603. 10.1038/nature13376 24847888

[5] Hansen MC, Potapov PV, Moore R, Hancher M, Turubanova SA, Tyukavina A, et al (2013) High-resolution global maps of 21st-century forest cover change. Science 342: 850–853. 10.1126/science.1244693 24233722

[6] Mitchard ETA, Saatchi SS, Lewis SL, Feldpausch TR, Woodhouse IH, Sonke B, et al (2011) Measuring biomass changes due to woody encroachment and deforestation/degradation in a forest–savanna boundary region of central Africa using multi-temporal L-band radar backscatter. Remote Sens Environ 115: 2861–2873.

[7] Hely C, Bremond L, Alleaume S, Smith B, Sykes MT, Guiot J (2006) Sensitivity of African biomes to changes in the precipitation regime. Global Ecol Biogeogr 15: 258–270.

[8] Bucini G, Hanan NP (2007) A continental-scale analysis of tree cover in African savannas. Global Ecol Biogeogr 16: 593–605.

[9] Torello-Raventos M, Feldpausch TR, Veenendaal E, Schrodt F, Saiz G, Domingues TF, et al (2013) On the delineation of tropical vegetation types with an emphasis on forest/savanna transitions. Plant Ecol Divers 6: 101–137.

[10] Boulvert Y (1990) Avancée ou recul de la forêt centroafricaine Changements climatiques, influence de l'homme et notamment de feux. In Paysages Quaternaries de l'Afrique Central Atlantique, eds. Lanfranchi R, Schwartz D (Initiations et Didactiques ORSTROM, Paris), 353–366.

[11] Bond WJ, Midgley GF (2012) Carbon dioxide and the uneasy interactions of trees and savannah grasses. Phil Trans R Soc B 367: 601–612. 10.1098/rstb.2011.0182 22232770 PMC3248705

[12] Lewis SL, Lopez-Gonzalez G, Sonke B, Affum-Baffoe K, Baker TR, Ojo LO, et al (2009) Increasing carbon storage in intact African tropical forests. Nature 457: 1003–1006. 10.1038/nature07771 19225523

[13] Lewis SL, Edwards DP, Galbraith D (2015) Increasing human dominance of tropical forests. Science 349: 827–832. 10.1126/science.aaa9932 26293955

[14] Chazdon RL (2003) Tropical forest recovery: legacies of human impact and natural disturbances. Perspectives in Plant Ecology, Evolution and Systematics 6: 51–71.

[15] Bonnell TR, Reyna-Hurtado R, Chapman CA (2011) Post-logging recovery time is longer than expected in an East African tropical forest. For Ecol Manage 261: 855–864.

[16] Gourlet-Fleury S, Mortier F, Fayolle A, Baya F, Ouédraogo D, Bénédet F, et al (2013a) Tropical forest recovery from logging: a 24 year silvicultural experiment from Central Africa. Phil Trans R Soc B 368: 20120302 10.1098/rstb.2012.0302 23878332 PMC3720023

[17] Gourlet-Fleury S, Beina D, Fayolle A, Ouédraogo DY, Mortier F, Bénédet F, et al (2013b) Silvicultural disturbance has little impact on tree species diversity in a Central African moist forest. For Ecol Manage 304: 322–332.

[18] White LJT (1995) Vegetation Study—Final Report: République du Gabon, Project ECOFAC- Composante Gabon, Libreville, Gabon: ECOFAC.

[19] White LJT (2001) Forest-savanna dynamics and the origins of Marantaceae forest in central Gabon In African rain forest ecology and conservation, eds. Weber W, White LJT, Vedder A, Naughton-Treves L (New Haven CT and London: Yale University Press) 165–182.

[20] Nana A (2005) Apport de la télédétection et du SIG pour le suivi de la dynamique forêt-savane. Cas au Gabon du Parc de la Lopé de 1982 à 1996. Libreville, University of Omar Bongo: DESS Thesis.

[21] Mitchard ETA, Saatchi SS, White LJT, Abernethy KA, Jeffery KJ, Lewis SL, et al (2012) Mapping tropical forest biomass with radar and spaceborne LiDAR: overcoming problems of high biomass and persistent cloud. Biogeosciences 9: 179–191.

[22] Zelazowski P, Malhi Y, Huntingford C, Sitch S, Fisher J (2011) Changes in the potential distribution of humid tropical forests on a warmer planet. Phil Trans R Soc A 369: 137–160. 10.1098/rsta.2010.0238 21115517

[23] Liénou G, Mahé G, Paturel J-E, Servat E, Sighomnou G, Ekodeck G, et al (2008) Evolution des régimes hydrologiques en région équatoriale camerounaise: un impact de la variabilité climatique en Afrique équatoriale? Hydrol Sci J 53: 789–801.

[24] Gond V, Fayolle A, Pennec A, Cornu G, Mayaux P, Camberlin P, et al (2013) Vegetation structure and greenness in Central Africa from Modis multi-temporal data. Phil Trans R Soc B 368: 20120309 10.1098/rstb.2012.0309 23878336 PMC3720027

[25] James R, Washington R, Rowell DP (2013) Implications of global warming for the climate of African rainforests. Phil Trans R Soc B 368: 20120298 10.1098/rstb.2012.0298 23878329 PMC3720020

[26] United Nations (2015) Paris Agreement of the United Nations Climate Change Conference, COP 21.

[27] Williams M, Ryan CM, Rees RM, Sambane E, Fernando J, Grace J (2008) Carbon sequestration and biodiversity in re-growing miombo woodlands in Mozambique. For Ecol Manage 254: 145–155.

[28] Gottschalk P, Smith JU, Wattenbach M, Bellarby J, Stehfest E, Arnell N, et al (2012) How will organic carbon stocks in mineral soils evolve under future climate? Global projections using RothC for a range of climate change scenarios. Biogeosciences 9: 3151–3171.

[29] Gourlet-Fleury S, Rossi V, Rejou-Mechain M, Freycon V, Fayolle A, Saint-André L, et al (2011) Environmental filtering of dense-wooded species controls above-ground biomass stored in African moist forests. J Ecol 99: 981–990.

[30] Quesada CA, Phillips OL, Schwarz M, Czimczik CI, Baker TR, Patiño S, et al (2012) Basin-wide variations in Amazon forest structure and function are mediated by both soils and climate. Biogeosciences 9: 2203–2246.

[31] Maley J (1996). The African rain forest–main characteristics of changes in vegetation and climate from the Upper Cretaceous to the Quaternary. P Roy Soc Edinb B 104: 31–73.

[32] White LJT, Abernethy K (1997) A guide to the vegetation of the Lope Reserve Gabon ECOFAC GABON, Libreville, Gabon.

[33] Walker BH (1981) Is succession a viable concept in African savanna ecosystems? In Forest Succession: Concepts and Application, eds. West DC, Shugart HH, Botkin DB (Springer New York), 431–447.

[34] UNEP (2009) Vital Forests report. Available: http://www.unep.org/vitalforest/Report/VFG-01-Forest-definition-and-extent.pdf. Accessed May 2016.

[35] IPCC (2000) Land Use, Land-Use Change and Forestry. ed. Watson RT, Noble IR, Bolin B, Ravindranath NH, Verardo DJ, Dokken DJ. Cambridge University Press, UK.

[36] Oslisly R, White LJT, Bentaleb I, Favier C, Fontugne M, Gilet J-F, et al (2013) Climatic and cultural canges in the west Congo basin forests over the past 5000 years. Philos Trans R Soc London 368: 20120304. 23878334 10.1098/rstb.2012.0304 PMC3720025

[37] Jeffery KJ, Korte L, Palla F, Walters G, White LJT, Abernethy KA (2014) Fire management in a changing landscape: a case study from Lopé National Park, Gabon. Parks 20: 35–48.

[38] Jupp DLB, Culvenor DS, Lovell JL, Newnham GJ, Strahler AH, Woodcock CE (2009) Estimating forest lai profiles and structural parameters using a ground-based laser called echidna. Tree Physiol 29: 171–181. 10.1093/treephys/tpn022 19203942

[39] Calders K, Newnham G, Burt A, Murphy S, Raumonen P, Herold M, et al (2015a) Nondestructive estimates of above-ground biomass using terrestrial laser scanning. Methods Ecol Evol 6: 198–208.

[40] Calders K, Armston J, Newnham G, Herold M, Goodwin N (2014) Implications of sensor configuration and topography on vertical plant profiles derived from terrestrial LiDAR. Agr Forest Meteorol 194: 104–117.

[41] Quesada CA, Lloyd J, Schwarz M, Patiño S, Baker TR, Czimczik C, et al (2010) Variations in chemical and physical properties of Amazon forest soils in relation to their genesis, Biogeosciences 7: 1515–1541.

[42] International Soil Reference and Information Centre (ISRIC) (2002) Procedures for Soil Analysis, technical paper 9, sixth edition, ed. van Reeuwijk LP. International Soil Reference and Information Centre, Wageningen, The Netherlands.

[43] Dohrmann R (2006) Cation exchange capacity methodology II: A modified silver–thiourea method. Appl Clay Sci 34: 38–46.

[44] Rowell DL (1994) Soil Science, Methods and Applications. Longman Group, UK.

[45] Tiessen H, Moir JO (1993) Characterisation of available P by sequential extraction In Soil sampling and methods of analysis, ed. Carter MR (Canadian Society of Soil Science, Lewis, Boca Raton, FL), 75–86.

[46] Roy DP, Boschetti L, Justice CO, Ju J (2008) The Collection 5 MODIS Burned Area Product—Global Evaluation by Comparison with the MODIS Active Fire Product. Remote Sens Environ 112: 3690–3707.

[47] Giglio L, Loboda T, Roy DP, Quayle B, Justice CO (2009) An active-fire based burned area mapping algorithm for the MODIS sensor. Remote Sens Environ 113:408–420.

[48] Lewis SL, Sonké B, Sunderland T, Begne SK, Lopez-Gonzalez G, van der Heijden GMF, et al (2013) Aboveground biomass and structure of 260 African tropical forests. Philos Trans R Soc London 368: 1896–1934. 10.1098/rstb.2012.0295 PMC3720018 23878327

[49] Chave J, Rejou-Mechain M, Burquez A, Chidumayo E, Colgan M S, Delitti WBC, et al (2014) Improved allometric models to estimate the aboveground biomass of tropical trees. Glob Change Biol 20: 3177–3190. 10.1111/gcb.12629 24817483

[50] Talbot J, Lewis SL, Lopez-Gonzalez G, Brienen RJW, Monteagudo A, Baker TR, et al (2014) Methods to estimate aboveground wood productivity from long-term forest inventory plots. For Ecol Manage 320: 30–38.

[51] Chave J, Coomes D, Jansen S, Lewis S, Swenson NG, Zanne AE (2009) Towards a worldwide wood economics spectrum. Ecol Lett 12: 351–366. 10.1111/j.1461-0248.2009.01285.x 19243406

[52] Zanne A, Lopez-Gonzalez G, Coomes DA, Ilic J, Jansen S, Lewis SL, et al (2009) Towards a Worldwide Wood Economics, Spectrum. 10.5061/dryad.234 19243406

[53] Feldpausch TR, Lloyd J, Lewis SL, Brienen RJW, Gloor M, Monteagudo Mendoza A, et al (2012) Tree height integrated into pantropical forest biomass estimates. Biogeosciences 9: 3381–3403.

[54] Bailey RL (1979) The potential of Weibull-type functions as flexible growth curves: discussion. Can J For Res 10: 117–118.

[55] Phillips OL, Gentry AH (1994) Increasing turnover through time in tropical forests. Science 263: 954–958. 17758638 10.1126/science.263.5149.954

[56] Calders K, Schenkels T, Bartholomeus H, Armston J, Verbesselt J, Herold M (2015b) Monitoring spring phenology with high temporal resolution terrestrial LiDAR measurements. Agr Forest Meteorol 203: 158–168.

[57] R Development Core Team (2013). R Development Core Team. R: A Language and Environment for Statistical Computing.

[58] Yerima BPK, van Ranst E (2005) Introduction to soil science: soils of the tropics Trafford Publishing, Victoria, Canada.

[59] Chiti T, Mihindou V, Jeffery KJ, Malhi Y, Oliveira F, White LJT, et al (in press) Impact of tropical forest succession on soil organic carbon storage in the Lopé National Park, Gabon. Biotropica

[60] Martin D (1981) Carte Pédologique du Gabon. Scale: 2,000,000. France.

[61] Coetsee C, Gray EF, Wakeling J, Wigley BJ, Bond WJ (2013) Low gains in ecosystem carbon with woody plant encroachment in a South African savannah. J Trop Ecol 29: 49–60.

[62] Djomo AN, Knohl A, Gravenhorst G (2011) Estimations of total ecosystem carbon pools distribution and carbon biomass current annual increment of a moist tropical forest. For Ecol Manage 261: 1448–1459.

[63] Coomes DA, Allen RB (2007) Mortality and tree-size distributions in natural mixed-age forests. J Ecol 95: 27–40.

[64] Coomes DA, Holdaway RJ, Kobe RK, Lines ER, Allen RB (2012) A general integrative framework for modelling woody biomass production and carbon sequestration rates in forests. J Ecol 100: 42–64.

[65] Fuhr M, Nasi R, Delegue M-A (2001) Vegetation structure, floristic composition and growth characteristics of *Aucoumea klaineana* Pierre stands as influenced by stand age and thinning. For Ecol Manage 140: 117–132.

[66] Coomes DA, Duncan RP, Allen RB, Truscott J (2003) Disturbances prevent stem size-density distributions in natural forests from following scaling relationships. Ecol Lett 6: 980–989.

[67] Devos C, Sanz C, Morgan D, Onononga JR, Laporte N, Huynen MC (2008) Comparing Ape densities and habitats in northern Congo: Surveys of Sympatric gorillas and chimpanzees in the Odzala and Ndoki Regions. Am J Primatol 70:1–13. 10.1002/ajp.20514 18176937

[68] Tovar C, Breman E, Brncic T, Harris DJ, Bailey R, Willis KJ (2014) Influence of 1100 years of burning on the central African rainforest. Ecography 37: 1139–1148.

[69] Pfeifer M, Burgess ND, Swetnam RD, Platts PJ, Willcock S, et al (2012) Protected Areas: Mixed Success in Conserving East Africa’s Evergreen Forests. PLoSONE 7(6): e39337. 10.1371/journal.pone.0039337 PMC3387152 22768074

[70] Guariguata MR, Ostertag R (2001) Neotropical secondary forest succession: changes in structural and functional characteristics. For Ecol Manage 148: 185–206.

[71] Brunck F, Grison F, Maıtre HF (1990) L’okoumé, *Aucoumea klaineana* Pierre. Monographie. CIRAD-CTFT, Nogent-sur-Marne, France.

[72] Peh KSH, Lewis SL, Lloyd J (2011) Mechanisms of monodominance in diverse tropical tree-dominated systems. J Ecol 99: 891–898.

[73] Martin AR, Thomas SC (2011) A Reassessment of Carbon Content in Tropical Trees. PLoS ONE 6(8): e23533 10.1371/journal.pone.0023533 21858157 PMC3157388

[74] de Wasseige C, Devers D, de Marcken P, Eba’a Atyi R, Nasi R, Mayaux P (2009) Les forêts du bassin du Congo: état des forêts 2008 Office des publications de l’Union européenne, Luxembourg.

[75] Engone Obiang NL, Ngomanda A, Hymas O, Chezeaux E, Picard N (2014) Diagnosing the demographic balance of two light-demanding tree species populations in central Africa from their diameter distribution. For Ecol Manage 313: 55–62.

[76] White LJT, Rogers ME, Tutin CEG, Williamson EA, Fernandez M (1995) Herbaceous vegetation in different forest types in the Lope Reserve, Gabon: implications for keystone food availability. Afr J Ecol 33: 124–141.

[77] Veenendaal EM, Torello-Raventos M, Feldpausch TR, Domingues TF, Gerard F, Schrodt F, et al (2015) Structural, physiognomic and above-ground biomass variation in savanna–forest transition zones on three continents–how different are co-occurring savanna and forest formations? Biogeosciences 12: 2927–2951.

[78] Mayaux P, Bartholome E, Fritz S, Belward A (2004) A new landcover map of Africa for the year 2000. J Biogeogr 31: 861–877.

[79] Njomgang R, Yemefack M, Nounamo L, Moukam A, Kotto-Same J (2011) Dynamics of shifting agricultural systems and organic carbon sequestration in southern Cameroon. Tropicultura 29: 176–182.

[80] Gautam S, Pietsch SA (2012) Carbon pools of an intact forest in Gabon. Afr J Ecol 50: 414–427. 23641117 10.1111/j.1365-2028.2012.01337.x PMC3636713

[81] Chiti T, Certini G, Grieco E, Valentini R (2010) The role of soil in storing carbon in tropical rainforests: the case of Ankasa Park, Ghana. Plant Soil 331: 453–461.

[82] Saiz G, Bird MI, Domingues T, Schrodt F, Schwarz M, Feldpausch TR, et al (2012) Variation in soil carbon stocks and their determinants across a precipitation gradient in West Africa. Glob Change Biol 18: 1670–1683.

